# Wild Small Mammals and Ticks in Zoos—Reservoir of Agents with Zoonotic Potential?

**DOI:** 10.3390/pathogens10060777

**Published:** 2021-06-21

**Authors:** Pavlína Pittermannová, Alena Žákovská, Petr Váňa, Jiřina Marková, František Treml, Lenka Černíková, Marie Budíková, Eva Bártová

**Affiliations:** 1 Department of Biology and Wildlife Diseases, Faculty of Veterinary Hygiene and Ecology, University of Veterinary Sciences Brno, Palackého tř. 1946/1, 61242 Brno, Czech Republic; pittermannova.p@gmail.com (P.P.); jirina.markova@gmail.com (J.M.); lenka.cernikova@svupraha.cz (L.Č.); 2 Department of Animal Physiology and Immunology, Faculty of Science, Masaryk University, Poříčí 7/9, 63900 Brno, Czech Republic; alenazak@sci.muni.cz (A.Ž.); pvana@mail.muni.cz (P.V.); 3 Department of Biology, Faculty of Education, Masaryk University, Poříčí 7/9, 63900 Brno, Czech Republic; 4 Department of Kinesiology, Faculty of Sports Studies, Masaryk University, Kamenice 753/5, 62500 Brno, Czech Republic; 5 Department of Infection Disease and Microbiology, Faculty of Veterinary Medicine, University of Veterinary Sciences Brno, Palackého tř. 1946/1, 61242 Brno, Czech Republic; tremlf@vfu.cz; 6 Department of Virology and Serology, State Veterinary Institute Prague, Sídlištní 136/24, 16503 Prague, Czech Republic; 7 Department of Mathematics and Statistics, Faculty of Science, Masaryk University, Kotlářská 2, 61137 Brno, Czech Republic; budikova@math.muni.cz

**Keywords:** *Anaplasma*, *Borrelia*, *Coxiella*, *Francisella*, *Leptospira*, *Rickettsia*

## Abstract

Wild small mammals and ticks play an important role in maintaining and spreading zoonoses in nature, as well as in captive animals. The aim of this study was to monitor selected agents with zoonotic potential in their reservoirs and vectors in a zoo, and to draw attention to the risk of possible contact with these pathogens. In total, 117 wild small mammals (rodents) and 166 ticks were collected in the area of Brno Zoo. Antibodies to the bacteria *Coxiella burnetii*, *Francisella tularensis*, and *Borrelia burgdorferi* s.l. were detected by a modified enzyme-linked immunosorbent assay in 19% (19/99), 4% (4/99), and 15% (15/99) of rodents, respectively. Antibodies to *Leptospira* spp. bacteria were detected by the microscopic agglutination test in 6% (4/63) of rodents. Coinfection (antibodies to more than two agents) were proved in 14.5% (15/97) of animals. The prevalence of *C. burnetii* statistically differed according to the years of trapping (*p* = 0.0241). The DNAs of *B. burgdorferi* s.l., *Rickettsia* sp., and *Anaplasma phagocytophilum* were detected by PCR in 16%, 6%, and 1% of ticks, respectively, without coinfection and without effect of life stage and sex of ticks on positivity. Sequencing showed homology with *R. helvetica* and *A. phagocytophilum* in four and one positive samples, respectively. The results of our study show that wild small mammals and ticks in a zoo could serve as reservoirs and vectors of infectious agents with zoonotic potential and thus present a risk of infection to zoo animals and also to keepers and visitors to a zoo.

## 1. Introduction

Wild small mammals (rodents) and ticks play an important role as reservoirs of agents causing zoonotic infections in wild animals, as well as in animals in captivity. The species of wild small mammals captured in this work (*Apodemus flavicollis*, *A. sylvaticus*, *Myodes glareolus*, *Sorex araneus*) belong to the most common rodents and insectivores found in natural environments in the Czech Republic, while *Mus musculus* and *Rattus norvegicus* are found frequently near human dwellings. Environments of zoos represent both the natural environment and human habitation. Wild small mammals in a zoo environment can participate in spreading several bacteria and thus pose a risk of infection to animals and humans. *Coxiella burnetii* and *Francisella tularensis* are two of the most dangerous and potentially very infectious aerosol-spreading bacteria causing outbreaks of disease. Other bacteria, *Leptospira* spp. and *Borrelia burgdorferi* s.l., are related in their structure but different in the way of infection. 

*Coxiella burnetii* bacteria cause Q fever. Humans and animals become infected through inhalation of contaminated dust or aerosol, ingestion of contaminated food, or skin injury during the handling of the tissue of infected animals [1]. Infection in humans is usually asymptomatic or manifests as a mild disease with spontaneous recovery; however, Q fever may lead to serious complications and even death in patients with acute disease of meningoencephalitis and myocarditis or in chronically infected patients with endocarditis [2]. *Francisella tularensis* bacteria cause tularemia. Humans and animals become infected through water contaminated with a rodent’s urine or feces, inhalation of contaminated aerosol, or skin injury during the handling of infected animals [3]. The most common form of the disease in humans is the ulceroglandular form, in which there is a painful sore at the site of the infection and a swelling of the lymph node that drains the area. Along with these local signs, the infected person has a fever that may persist for 2 or 3 weeks, with headache, vomiting, body pains, and general weakness. Infection of the eye is also common with swelling of related lymph glands [4]. Pathogenic representants of *Leptospira* spp. bacteria (especially *Leptospira interrogans* sensu lato) cause leptospirosis. Humans and animals become infected after exposure to animal urine directly or via contamination of soil or water through damaged skin or via the mucous membranes of the nose, mouth, or eyes. These bacteria are characterized by natural nidality. Each serovar tends to be maintained in specific reservoir hosts [5] (e.g., rats are typical reservoir hosts of *icterohaemorrhagiae* and *copenhageni* serovars, while mice are reservoir hosts of *ballum*, *arborea*, and *bim* serovars) [6]. The initial symptoms of leptospirosis are flulike: fever, chills, headache, nausea, vomiting, cough, and diarrhea, and some patients may also have red eyes (conjunctival suffusion). Many patients have a mild illness or no symptoms at all, but about 10% become severely ill with jaundice (yellow eyes or skin) due to liver dysfunction and significant bleeding. That is why they may require dialysis for kidney failure or a ventilator for respiratory failure. The most severe form of leptospirosis is called Weil’s disease [7].

Bacterial infection in a zoo environment can also be spread through vectors. The most common vectors of tick-borne diseases (Lyme diseases, rickettsiosis, and anaplasmosis) in the northern hemisphere are ticks of the *Ixodes ricinus* complex [8]. *Borrelia burgdorferi* (sensu lato) bacteria cause Lyme disease. In Europe, at least five different genotypes (*B. afzelii*, *B. bavariensis, B. burgdorferi sensu stricto*, *B. garinii*, and *B. spielmanii*) are pathogenic to humans [9]. Humans usually become infected through the bite of infected ticks; therefore, the occurrence of this disease in humans depends on the population density of infected ticks and mainly on infected nymphal ticks [10]. The variation in the density of nymphs is associated with the density of rodents in the previous year [8] because a higher density of rodents gives rise to the opportunity in larval stages of ticks for the feeding and development of nymphs in the following year. The danger of Lyme diseases is that a typical red spot of erythema migrans is not formed in 40%–60% of infected humans, and after that, the infection can progress to a very complicated chronic state [11]. Among the four groups of *Rickettsia* bacteria, only spotted fever group rickettsiae cause a tick-borne disease [12] that develops after the bite of infected ticks. The disease in humans is characterized by generic flulike symptoms or lymphadenopathies with or without a consistent rash and sometimes an eschar (“tache noire”) at the site of the tick bite. Symptoms are usually mild, but sometimes can be severe or even lethal in humans with predisposing conditions associated with some diseases (e.g., diabetes, alcoholism, or cirrhosis) [13]. *Anaplasma phagocytophilum* bacteria, with zoonotic potential, are transmitted through the bite of infected ticks, and small mammals (mice, voles, shrews, and hedgehogs) can serve as a reservoir of infection [14]. A disease caused by these bacteria is human granulocytic anaplasmosis (HGA) with hematologic abnormalities, including marked leukopenia and thrombocytopenia. Although the immune mechanisms that account for severe and fatal HGA are not completely understood, there is some evidence of immunosuppression in patients with HGA, which is supported by the high number of fatal cases due to secondary opportunistic infections and organ failures [15].

Zoological gardens are institutions with a large concentration and variety of animals requiring highly specific care. The animals are gathered in a relatively small area, which manifests the increased risk of outbreaks of diseases, including zoonosis. Different infectious agents can be transmitted from reservoirs and vectors (wild small mammals and ticks) to zoo animals, but also to keepers and zoo visitors. Nevertheless, the monitoring of infectious agents with zoonotic potential in zoological gardens is neglected. That is why the aim of the study was to monitor selected infectious agents in wild small mammals and ticks collected in Brno Zoo and to evaluate the possible risk of zoonotic infection to animals and humans. 

## 2. Results

Antibodies to *C. burnetii* were detected in 19% (19/99) of wild small mammals. To our knowledge, this is the first study focusing on the detection of *C. burnetii* antibodies in the area of a zoo in the Czech Republic. The seropositivity of *C. burnetii* statistically differed in the years of animal trapping (*p* = 0.0241), while other factors (species, sex, and year of rodents) had no effect on the seropositivity. Antibodies to *F. tularensis* and *B. burgdorferi* were detected in 4% (4/99) and 15% (15/99) of animals, respectively, without statistical differences in any of the observed factors. Antibodies to *Leptospira* spp. (serovars *L. grippotyphosa*, approximately 1.6%; *L. icterohaemorrhagiae*, 1.6%; and *L. sejroe*, 3.2%) were detected in 6.3% (4/63) of animals with titers 200, 400, 1600, and 1600 in one *A. flavicollis* (*L. grippotyphosa*), two *Mus musculus* (*L. sejroe*), and one *Rattus norvegicus* (*L. icterohaemorrhagiae*). Antibodies to more than two agents were proved in 14.5% (15/97) of animals, showing that during life animals are exposed to more infections. The positive animals had statistically different (*p* < 0.0001) types of antibodies (IgM and IgG). In the case of *C. burnetii* and *F. tularensis*, more animals had IgM antibodies typical for acute infection, while in the case of *B. burgdorferi* s.l., more animals had IgG antibodies typical for chronic infection. The results obtained from wild small mammals are summarized in Table 1.

**Figure 1:**
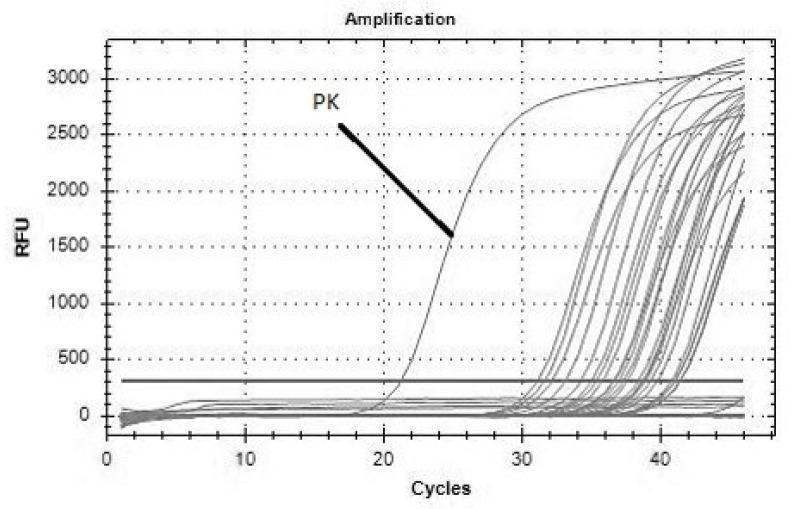
Detection of *B. burgdorferi* s.l. by RT-PCR in ticks collected from Brno Zoo. RFU—relative fluorescence unit, cycles—number of cycles in real-time PCR, PK—positive control of *B. burgdorferi* s.l.

**Figure 2:**
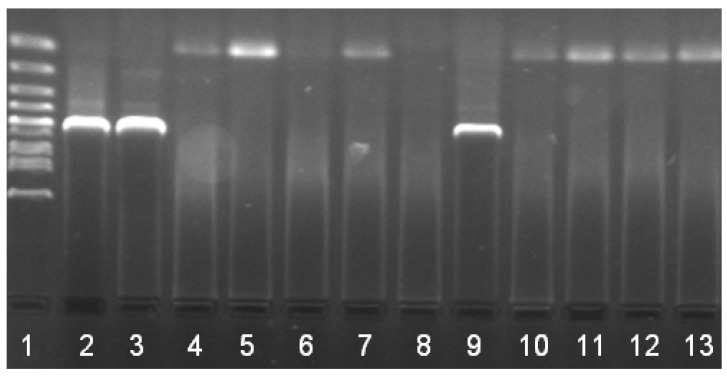
Detection of *Anaplasma phagocytophilum* by PCR in ticks collected from Brno Zoo: 1 standard (100 bp); 2, 3 positive controls; 4–12 tested samples with 1 positive sample (number 9; size of band 500 bp); 13 negative control.

**Figure 3:**
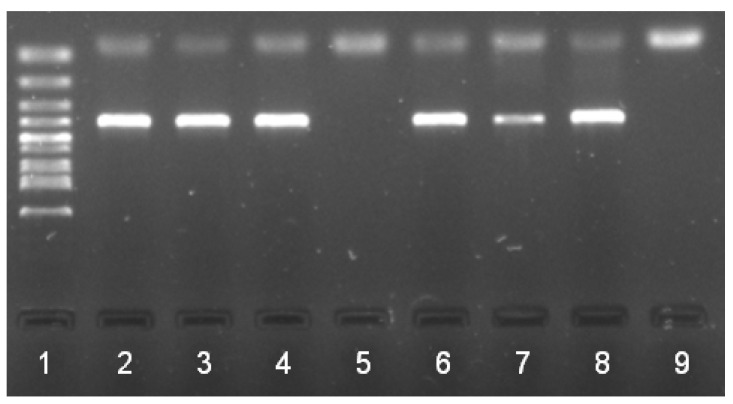
Detection of *Rickettsia* sp. by PCR in ticks collected from Brno Zoo: 1 standard (100 bp); 2, positive control; 3–8 tested samples with five positive samples (numbers 3, 4, 6, 7, 8; size of band 401 bp); 9 negative control.

The DNAs of *B. burgdorferi* s.l., *Rickettsia* sp., and *A. phagocytophilum* were detected by PCR in 16% (11/70), 6% (4/70), and 1% (1/70) of ticks (Figure 1, Figure 2 and Figure 3 showing examples of the PCR results), respectively, without coinfection and without effect of life stage and sex of ticks on positivity (*p* > 0.05). Five samples positive in PCR were successfully sequenced, showing the homology with *Rickettsia helvetica* (*n* = 4) and *A. phagocytophilum* (*n* = 1). The results obtained from ticks are summarized in Table 2.

In wild small mammals and ticks, different methods (serology and PCR, respectively) were used to detect infectious agents; nevertheless, nearly the same positivity (15% and 16%, respectively) was obtained for *B. burgdorferi* s.l. 

## 3. Discussion

A zoo has a diverse collection of exotic animals living in a small area, where they can be easily exposed to different infectious agents. Wild rodents present in the area of a zoo can be involved in the spread of infections, since they are the most common hosts of ticks (*I. ricinus*) and reservoirs of tick-borne pathogens causing zoonotic diseases. 

Antibodies to *C. burnetii* were detected by ELISA in 19% of rodents, with the highest prevalence in *A. sylvaticus* and *R. norvegicus*. A similar result (12%) was obtained from wild small mammals from three Moravian natural localities in the Czech Republic [16], with the highest prevalence in *M. glareolus*. In the case of *C. burnetii*, the positivity statistically differed in the years of trapping (*p* = 0.0241). The higher prevalence in 2017 (41%), compared with 21% and 11% in 2016 and 2018, respectively, could be explained by the transport of 55 new animals (38 species of vertebrates and 17 species of invertebrates) from other Czech and European zoos to Brno Zoo in 2017 (personal communication with Brno Zoo vet). Pathogenic *C. burnetii* also circulates in the wild in other European countries. In the United Kingdom, antibodies to *C. burnetii* were detected in 17% of 796 rodents, including the same animal species (*M. glareolus* and *A. sylvaticus*) found in this study [17]. In Poland, *C. burnetii* was detected by PCR in tissues of 3% of wild-living animals (deer, roe deer, and boar) [18]. In Slovakia, antibodies to *C. burnetii* were detected by ELISA in 30% of ruminants (4 mouflons, 60 fallow deer, 9 Cameroon goats, 8 Carpathian goats, and 8 Cameroon sheep) from a zoo [19]. The high infection rate of *C. burnetii* antibodies indicates a similar result as in our study. To our knowledge, there is no published study focusing on the detection of *C. burnetii* in wild small mammals in a zoo area. 

Antibodies to *F. tularensis* were detected in 4% of wild small mammals in Brno Zoo. In Germany, Otto et al. [20] tested 2475 animals (wild foxes, raccoon dogs, and wild boars) from a zoo for antibodies to *F. tularensis* with a prevalence of 1.1% for wild boars and 7.4% for red foxes. They used a similar technique of indirect ELISA, and in addition, positive and inconclusive samples were confirmed by Western blot. Other studies in Europe are mainly based on the use of the methods of molecular biology and related to animals trapped in the wild. For example, *F. tularensis* was detected by PCR in 0.9% out of 547 wild small mammals from Finland, with only one positive species, *M. agrestis* [21], and in 1.4% of wild rodents from Italy [22]. On the other hand, negative results were obtained by both RT-PCR/PCR and serological methods in 110 wild rodents (*M. glareolus, A. flavicollis, A. sylvaticus*, and *M. arvalis*) trapped in the region of Lower Austria [23]. 

The results of our study show evidence that *F.* *tularensis* is circulating in zoos and thus poses the risk of infection to animals and humans, especially considering that there are a higher number of human patients with tularemia in the Czech Republic compared with other European countries. More specifically, the Czech Republic is among the countries with the highest incidence of tularemia, with an average of 55 patients with tularemia per year and 0.55 per 100,000 inhabitants in the last 10 years, while the number of patients in 2019 increased threefold [24]. In contrast, there were only seven cases of Q fever reported in the Czech Republic in the last 10 years [24]. In our study, we obtained opposite results, with a higher seroprevalence of *C. burnetii* (19%) compared with *F. tularensis* (4%). In Austria, a neighboring country of the Czech Republic, no case of tularemia has been reported, and its notification rate is 0 cases per 100,000 inhabitants. In contrast, the highest notification rate has been reported in Sweden (1.56 cases per 100,000 inhabitants). 

The seroprevalence of *B. burgdorferi* s.l. (15%) obtained from rodents from a zoo was in the range of the seroprevalence (12–44%) obtained from rodents trapped in natural localities of the Czech Republic with the use of the same method [25,26,27]. In Slovakia, the seroprevalence obtained by modified ELISA in wild small mammals corresponded (19%) with our results [28] or was lower (10%) [29]. *B. burgdorferi* s.l. was also detected by PCR in 16% [26] and 10% [27] of wild rodents in the Czech Republic. The results of a study performed in five Czech zoos showed the presence of *B. burgdorferi* s.l. antibodies in 60% of zoo animals (including ungulates, carnivores, primates, birds, and reptiles) detected by LYMETOP VET+, a rapid qualitative commercial immunochromatographic test [30]. Such a high seropositivity in zoo animals may indicate the presence of infectious agents in the zoo area and the transmission from vectors (ticks) and intermediate hosts (rodents). A lower prevalence (10%) of *B. burgdorferi* s.l. antibodies was detected by ELISA in ungulates and carnivores from 11 zoos and parks in Germany [31]. 

The seroprevalence of antibodies to *Leptospira* spp. (serovars *L. grippotyphosa*, approximately 1.6%; *L. icterohaemorrhagiae*, 1.6%; and *L. sejroe*, 3.2%) in wild small mammals trapped in Brno Zoo was 6%. This prevalence is the same as the prevalence (5.4%) detected in wild small mammals (*n* = 2113) trapped in Eastern Slovakia between 1991 and 1995 [32]. They reported antibodies to *Leptospira* spp. in five rodent species (*A. flavicollis*, *A. agrarius*, *A. microps*, *Clethrionomys glareolus*, and *Microtus arvalis*), with the most frequently observed serovars *L. grippothyphosa* (65%) and *L. sejroe* (26%). The global seroprevalence of *Leptospira* in wild rats (*Rattus* spp.), which are the most important sources of *Leptospira* infection, varies widely from 0% to 73.7% [33]. On the other hand, another small mammal, ground squirrel (*Spermophilus beecheyi*), also considered to be a reservoir of diseases in zoos, exhibited 57% (*n* = 42) seropositivity for various *L. interrogans* serovars when tested in Wildlife Safari, a wild animal park in Winston, Oregon, USA [34]. The prevalence was much higher compared with our study, even though the monitoring of seroprevalence in the squirrel population was done during a hot and dry summer in contrast to the summer in Brno, which is not so hot and dry. It indicates that various environmental factors may play an important role in the epidemiology of this pathogen. In zoos, the population density of both captive animals and reservoirs (vectors), environmental factors (including the natural nidality of pathogens), state of health of captive animals, and spatial relationships between captive and wild animals play an important role in the epidemiology of this pathogen [35,36]. 

In the case of bacterial DNA isolated from ticks, *B. burgdorferi* s.l. (15.7%) displayed the highest prevalence, followed by *Rickettsia* sp. (5.7%) and *A. phagocytophilum* (1.4%). The level of tick positivity for *B. burgdorferi* s.l. is consistent with the seroprevalence obtained from rodents trapped in the zoo. The level of positivity obtained from ticks from the Czech Republic varies and depends on the localities with 12%–21% positivity in ticks from South Bohemia [36,37] and 6.4% in those from South Moravia [38]. The lower prevalence (10.2%) of *B. burgdorferi* s.l. was detected by molecular methods in ticks from Eastern Slovakia [29]. The lower positivity of *Rickettsia* sp. and *A. phagocytophilum*, shown in this study, compared with *B. burgdorferi* s.l., has also been reported in other studies. Venclikova et al. [39] examined ticks in natural and urban ecosystems using molecular methods with a minimum infection rate (MIR) for *Rickettsia* spp. of 2.9% in urban parks and 3.4% in natural forest ecosystems in the Czech Republic, while the MIRs for *A. phagocytophilum* were 9.4% and 1.9%. On the other hand, Hönig et al. [40] detected *Rickettsia* spp. and *A. phagocytophilum* in 3.9% and in 2.8% of *I. ricinus* ticks collected from the Southern Czech Republic. Moreover, Svitálková et al. [41] monitored *A. phagocytophilum* prevalence in *I. ricinus* and rodents in various habitat types of Slovakia by real-time PCR, with a significantly higher prevalence of *A. phagocytophilum* in *I*. *ricinus* from the urban/suburban habitat (7.2%) compared with that from the natural habitat (3.1%) in Southwest Slovakia. Their results suggest that rodents are not the main reservoirs of the bacterium in the investigated area. To our knowledge, there are no similar studies performed on ticks from a zoo. 

## 4. Materials and Methods

### 4.1. Sampling (Wild Small Mammals, Ticks)

Sampling was performed in Brno Zoo between 2016 and 2018. The zoo is located on the outskirts of the city of Brno (coordinates: 49°13′49.6″ N, 16°31′59.9″ E) near a dam. It covers an area of almost 65 ha, with an exposition area of 25 ha. The zoo houses approximately 408 animal species of mammals, birds, reptiles, amphibians, fish, and invertebrates. Wild small mammals were trapped by using spring traps and life-hunt traps inside pavilions (for exotic birds, tapirs, and terraria animals), in the technical room, and outside. In total, 117 wild small mammals of 6 animal species were trapped: yellow-necked field mouse (*Apodemus flavicollis*), long-tailed field mouse (*A. sylvaticus*), brown rat (*Rattus norvegicus* var. *alba*), bank vole (*Myodes glareolus*), house mouse (*Mus musculus*), and common shrew (*Sorex araneus*). The animals were dissected to collect tissue samples (heart, brain, and liver) for further examination. Hearts were individually put into 500 µL of 0.85% phosphate-buffered saline at 4 °C. After 1 day, the heart was removed, the heart rinse was centrifuged, and the supernatant was stored at −18 °C. Brain and liver tissues were stored at −20 °C and used for another research. 

Ticks were collected by flagging along the sidewalks where zoo visitors move, around the children’s playground, and close to the enclosures of llamas, horses, and birds. In total, 166 ticks (*Ixodes ricinus*) were collected and divided into 70 samples according to sex and developmental stage, as males, females, and nymphs (5 nymphs were joined into 1 sample). 

The researchers involved in the sampling had experience in safety measures during animal trapping and handling, and they followed the conditions of the long-term experimental project (see data availability statement). Basic principles of biosafety to protect persons and the environment were respected. The animals were necropsied in the isolated zone, and due to the possible risk of transmission of the pathogenic agents from the animals, the researchers used laboratory coats, gloves, eye and face protections, and respirators (3M). 

### 4.2. Serology

Antibodies to *C. burnetii*, *F. tularensis*, and *B. burgdorferi* s.l. were detected in heart rinses of wild small mammals by the enzyme-linked immunosorbent assay (ELISA) used for the detection of *B. burgdorferi* s.l. antibodies (TestLine, Prague, Czech Republic). The only modification of ELISA was the usage of a mixture of ultrasonically disrupted whole-cell antigens of *C. burnetii* (Batch No. 87, Virological Institute Academy of Sciences, Bratislava, Slovakia) and *F. tularensis* (Bioveta, a.s., Ivanovice na Hané, Czech Republic) and a mixture of three subspecies of *B. burgdorferi* s.l. (*B. afzelii* BRZX27 MSLB 8065, *B. garinii* BRZX 23 MSLB 8064, and *B. burgdorferi* s.s. WSLB 8014/1) [16,25]. The heart rinses were diluted according to the protein concentration of 100× diluted sera. The goat anti-mouse IgM and IgG conjugates (Sigma-Aldrich spol. s.r.o., Prague, Czech Republic) were used. Positive controls were prepared by immunization of BALB/c mice with 300 μL of the above-mentioned mixture of *B. burgdorferi* s.l. antigens, while C. *burnetii* and *F. tularensis* (40 μg/mL of antigen mixed with 1 mg/mL aluminum hydroxide in 0.85% physiological solution) were injected separately according to Žákovská et al. [42]. The antigens used in ELISA and for the immunization of mice to obtain positive controls were the same. A serum of wild mouse negative to *C. burnetii*, *F. tularensis*, and *B. burgdorferi* s.l. served as negative control. Absorbance of samples was measured at 492 nm by a spectrophotometer (SLT RainBow, Schoeller Instruments s.r.o., Prague, Czech Republic); the OD of IgM and IgG positive controls had an approximate value of 1.0. Samples were marked as positive in the case of IgM or IgG antibodies or both being detected. 

Antibodies to *Leptospira* spp. were detected in the heart printing (blood on the surface of the heart printed on a piece of filter paper) of wild small mammals by the microscopic agglutination test (MAT). Samples were marked as positive when ≥50% of visible leptospiras appeared to be agglutinated [43]. In total, 8 serovars of *Leptospira* (*L. grippotyphosa*, *L. icterohaemorrhagiae*, *L. bratislava*, *L. canicola*, *L. sejroe*, *L. sorex-jalna*, *L. pomona*, and *L. pyrogenes*) (Bioveta, a.s.), representing the serogroups Grippotyphosa, Icterohaemorrhagiae, Australis, Canicola, Sejroe, Javanica, Pomona, and Pyrogenes, respectively, were used. Samples with titers ≥100 were marked as positive.

### 4.3. Molecular Methods

DNA was isolated from ticks with NucleoSpin Tissue (Macherey-Nagel, Düren, Germany) to detect *B. burgdorferi* s.l. by real-time PCR using the primers p16Swt-fwd and p16Swtrev to amplify 16S rRNA [44], to detect *Rickettsia* sp. by single PCR using the primers CS 78 and CS 323 to amplify the gene gltA [45], and to detect *A. phagocytophilum* by nested PCR using two sets of the primers ge3a, ge10r and ge9f, ge2 to amplify gene 16S rRNA [46]. Samples positive for *Rickettsia* sp. were purified and confirmed by Sanger sequencing (Macrogen Europe B.V., Amsterdam, The Netherlands). 

### 4.4. Statistical Analysis

Data analysis was performed with the Pearson chi-square test for independence in a contingency table using Statistica Cz 12 [47]. Fisher’s exact test was used only for 2 × 2 tables if the conditions of good approximation were not met. We tested the null hypothesis that the seroprevalence of bacterial infection in wild small mammals does not differ by animal species, sex, age, and year of trapping and that the seroprevalence of bacterial infection in ticks does not differ by life stage and sex. McNemar’s test was used to compare IgM and IgG prevalences for *C. burnetii*, *F. tularensis*, and *B. burgdorferi*. The null hypothesis that the probability of the occurrence of IgM antibodies is the same as the probability of the occurrence of IgG antibodies was tested. The differences were considered statistically significant when the *p*-value was ≤0.05. Cluster analysis was used for the statistical evaluation of ELISA samples positive, dubious, and negative for IgM and IgG antibodies. Data showed normal distribution based on the Shapiro–Wilk and Kolmogorov–Smirnov tests. 

## 5. Conclusions

Information on the incidence, distribution, and risk to humans of pathogenic microorganisms coming from rodents and ticks in a zoo is limited. In our study, we confirmed that infectious agents with zoonotic potential are circulating in zoos, so animals and humans (keepers and visitors) may be at risk of infection. Regarding the direct detection of three zoonotic pathogens in ticks, the results were comparable to those obtained in recent years from studies carried out in natural locations in the Czech Republic. Even though zoo animals may be potential reservoirs of *C. burnetii* and *F. tularensis*, which are highly contagious pathogens, to our knowledge, none of the staff, veterinarians, or visitors have suffered from Q fever or tularemia.

## Figures and Tables

**Table 1:** Detection of antibodies to *Coxiella burnetii*, *Francisella tularensis*, and *Borrelia burgdorferi* s.l. by the enzyme-linked immunosorbent assay (ELISA) and *Leptospira* spp. by the microscopic agglutination test (MAT) in wild small mammals trapped in Brno Zoo.

Characteristic	Total	Serology									
		*C. burnetii*			*F. tularensis*			*B. burgdorferi* s.l.			*Leptospira* spp.
		Total	IgM	IgG	Total	IgM	IgG	Total	IgM	IgG	Total
**Species**											
*Apodemus flavicollis*	44	6/37 (16%)	6 (16%)	0 (0%)	2/37 (5%)	2 (5%)	0 (0%)	3/37 (8%)	0 (0%)	0 (0%)	1/17 (6%)
*Apodemus sylvaticus*	20	4/10 (40%)	4 (40%)	0 (0%)	1/10 (10%)	1 (10%)	0 (0%)	2/10 (20%)	0 (0%)	1 (10%)	0/1
*Mus musculus*	40	7/40 (18%)	5 (13%)	2 (5%)	1/40 (3%)	0 (0%)	1 (3%)	10/40 (25%)	2 (5%)	6 (15%)	2/35 (6%)
*Myodes glareolus*	4	0/3	0	0	0/3	0	0	0/3	0	0	0/2
*Rattus norvegicus* var. *alba*	6	2/6 (33%)	2 (33%)	0 (0%)	0/6 (0%)	0 (0%)	0 (0%)	0/6 (0%)	0 (0%)	0 (0%)	1/6 (17%)
*Sorex araneus*	3	0/3	0	0	0/3	0	0	0/3	0	0	0/2
**Sex**											
Female	58	8/52 (15%)	8 (15%)	0 (0%)	3/52 (6%)	2 (4%)	1 (2%)	7/52 (13%)	0 (0%)	3 (6%)	1/38 (3%)
Male	59	11/47 (23%)	9 (19%)	2 (4%)	1/47 (2%)	1 (2%)	0 (0%)	8/47 (17%)	2 (4%)	4 (9%)	3/25 (12%)
**Age**											
Adult	60	9/51 (18%)	7 (14%)	2 (4%)	2/51 (4%)	2 (4%)	0 (0%)	8/51 (16%)	2 (4%)	4 (8%)	0/32 (0%)
Juvenile	57	10/48 (21%)	10 (21%)	0 (0%)	2/48 (4%)	1 (2%)	1 (2%)	7/48 (15%)	0 (0%)	3 (6%)	4/31 (13%)
Year of trapping *											
2016	29	6/29 (21%)	6 (21%)	0 (0%)	2/29 (7%)	2 (7%)	0 (0%)	2/29 (7%)	0 (0%)	0 (0%)	1/10 (10%)
2017	35	7/17 (41%)	7 (41%)	0 (0%)	1/17 (6%)	1 (6%)	0 (0%)	3/17 (18%)	0 (0%)	2 (12%)	0/0
2018	53	6/53 (11%)	4 (8%)	2 (4%)	1/53 (2%)	0 (0%)	1 (2%)	10/53 (19%)	2 (4%)	5 (9%)	3/53 (6%)
**Total**	117	19/99 (19%)	17 (17%)	2 (2%)	4/99 (4%)	3 (3%)	1 (1%)	15/99 (15%)	2 (2%)	7 (7%)	4/63 (6%)

* The only statistical difference was in the prevalence of *C. burnetii* in years of trapping (*p* = 0.0241).

**Table 2:** Detection of *Borrelia burgdorferi* s.l., *Rickettsia* sp., and *Anaplasma phagocytophilum* by polymerase chain reaction (PCR) in ticks collected from Brno Zoo.

Characteristic	Total	PCR		
		*B. burgdorferi* s.l.	*Rickettsia* sp.	*A. phagocytophilum*
**Life stage**				
Nymph	24	6/24 (25%)	3/24 (12.5%)	0/24
Adult	46	5/46 (10.9%)	1/46 (2.2%)	1/46 (2.2%)
**Sex**				
Female	21	2/21 (9.5%)	0/21	1/21 (4.8%)
Male	25	3/25 (12%)	1/25 (4%)	0/25
**Total**	70	11/70 (16%)	4/70 (6%)	1/70 (1%)

## Data Availability

The data that support the findings of this study are available on request from the corresponding author. The data are not publicly available due to privacy or ethical restrictions.
